# Medical and Surgical Practice during the Santiago Campaign

**Published:** 1899-05

**Authors:** Wm. F. James

**Affiliations:** San Antonio, Texas


### TEXAS MEDICAL JOURNAL.

ESTABLISHED JULY, 1885.

PUBLISHED MONTHLY.—SUBSCRIPTION $1.00 A YEAR.

VOL. XIV. AUSTIN, MAY, 1899. No. 11.

### Original Contributions.

For Texas Medical Journal.

### Medical and Surgical Practice During the Santiago Campaign.

BY WM. F. JAMES, M. D., SAN ANTONIO, TEXAS.

MR. PRESIDENT AND GENTLEMEN: Before touching upon the medical and surgical practice during the Santiago campaign, I would like to say a few words as to the make-up of a regimental hospital corps, and also as to the conditions under which we had to work.

Each regiment is allowed three surgeons, two hospital stewards, and four hospital privates. The bearers are detailed from the troops during action. If the regiment has a band, the bandsmen usually act as bearers. The medical and surgical supplies consist of a large medical chest in which almost all the medicines are in tablet form, a large surgical chest containing instruments, antiseptic dressings and solutions, etc. Each doctor, steward and private also has pouches in which to carry dressings on the field.

The country was a very difficult one for an army to work in. There are absolutely no improvements, and the roads are in the worst possible condition. If you leave them, you usually plunge at once into a dense thicket.

Immediately upon landing we encountered our first difficulty.

Read at the 22nd annual meeting of the West Texas District Medical Association, San Antonio, April, 1899.

We applied for transportation for our medical supplies and dressings, and were told that there was none. Our medical officers tried by every means possible to get transportation, but found that it was useless, and that every other regiment was in the same condition. Those in authority did not seem to care whether we got any medical supplies to the front or not.

During the first three weeks after landing, the Rough Riders were the only regiment that had its medical and surgical chests at the front, and we never failed to have them open and have some sort of a hospital rigged up at each stopping place. We accomplished this by just rustling for ourselves. The first few days we used an officer's horse as a pack animal. Then one day I found an American horse in the brush, wounded in the leg, from which I extracted a shrapnell bullet, and in a few days he was well and made a splendid pack animal. Soon after that a pack mule got loose from a train and came straying around camp; he was promptly caught and pressed into service, so now we were well fixed as to transportation.

The division hospital was situated on the road to the coast, about five miles behind our lines at San Juan, and the village of Siboney, on the coast, where the hospital ships were lying, was about eight miles behind that again. Each regiment was supposed to have its dressing station as close behind its fighting line as it could consistently with safety. One regimental surgeon would stay at the dressing station, and the others would be right behind the firing line, so as to be able to give immediate "first aid" to the wounded. These each carried a pouch of surgical dressings and a sharp jack knife. As soon as a man was wounded, the clothes over the wound was slit up and compresses of sublimate gauze applied and tightly bandaged. He was then placed in the shade and as safe a place as possible, and left. Here again the transportation facilities were woefully lacking. There were not nearly enough ambulances, and we had only two stretchers for our whole regiment. Everything had to be improvised. Those who could walk with some assistance, were helped to the rear, others were carried, and most uncomfortable stretchers were improvised out of army blankets for the worst cases. Quite a number of men, I am afraid, fell in those thickets who never were found, and in many cases it was days before badly wounded men reached the division hospital.

Each man carried in the breast pocket of his shirt a first aid package of antiseptic dressings in the use of which he had been previously instructed. I think these packages of dressings must have saved many lives, and were very effective. They contained:

(1) Two antiseptic compresses of sublimate gauze in oiled paper.

(2) One antiseptic bandage of sublimated cambric with safety pin; and

(3) One large triangular bandage with safety pin.

Upon this triangular bandage were several illustrations showing the manner of its application to different parts of the body.

I took off these first dressings from wounds on which they had been placed three and four days previously, and the wounds were in fairly good condition. It is a very rare thing to see a wound in a septic condition. We had an abundance of antiseptic dressings and solutions, and they were freely used.

In past wars a cartridge has always been used carrying a leaden bullet. In this war the Spanish, as well as ourselves, used a bullet coated with nickel steel. This bullet has a long range—2000 yards—a very high velocity, and great penetrating power. It will pierce a one-half inch steel plate, or three feet of solid pine. An ordinary tree two feet thick is no protection whatever. The difference in effect between it and the old leaden bullet may be summed up as follows:

*The leaden bullet has—*

(1.) Shorter range.
(2.) Less velocity.
(3.) Less penetrating power.
(4.) On hitting the body it mushrooms out making a large wound at exit.
(5.) On hitting a long bone it usually fractures it for six or eight inches.
(6.) It almost invariably remains in the body.
(7.) It leaves a suppurating wound which is a long time in healing.
(8.) And when a long bone is struck, amputation of the limb is almost surely necessary.

*The nickel steel bullet has—*

(1.) A long range.
(2.) Very high velocity.
(3.) Great penetrating power.
(4.) On hitting the body it usually goes clean through the part without in the least altering its shape.
(5.) The aperture of entrance can almost be covered with the end of a lead pencil, and the aperture of exit is very slightly larger.

(6.) On hitting a large bone it bores a clean hole through it without fracturing it, unless the diameter of the bone is only slightly larger than the diameter of the bullet.

(7.) It rarely, unless nearly spent, remains in the body.

(8.) Wounds from it heal very kindly and quickly, with scarcely any suppuration.

(9.) And amputation of a limb from its effects is very rarely necessary.

The Mauser bullet is quite long, being about one and one-fourth inches, and sometimes, when it is nearly spent, it will make an ugly wound by turning sideways. The Spaniards also used an explosive bullet. This cartridge was about the size of our Springfield, and carried a leaden bullet covered by a brass jacket. This brass jacket would fly off from the lead and make a badly lacerated wound.

The most horrible and hopeless wounds were made by the shrapnell. On one of them bursting, pieces of scrap iron, brass, copper and round bullets would fly in every direction. A small piece of shrapnell shell would carry away a whole limb and maim and lacerate in the most horrible manner. The shrapnell bullets would invariably remain in the body. There was no fighting at close quarters, and I saw no sword or bayonet wounds.

On the whole, I have come to the conclusion that the surgery of the war was a success. Antiseptics played a prominent part, amputations were very rare, and the mortality from wounds very low. Of these modern rifles, I think it can be said that unless you were hit in some vital spot, the chances were everything in favor of your ultimate recovery.

Of the medical practice, I am sorry to say I cannot speak so well. In the first place, nearly every drug was in tablet form, and their administration and action was not at all satisfactory. Then again, there was altogether too much routine, and too little time and care bestowed upon diagnosis. The men, when they enlisted, were of course in splendid physical condition. The first thing to prostrate them after landing in Cuba was the intense tropical heat. On the marches they would fall out by the dozen, some of them dropping insensible. During the first week in July the men suffered terribly in the trenches. These trenches were frequently half full of water, there was absolutely no protection from the sun, very little food, and the men were constantly under an intense nervous strain, day and night, expecting attack. During that first week, too, there was very little rest to be had, as the trenches had to be dug and enlarged at night. This soon began telling upon the men, and each day more and more appeared at sick call, and after the surrender of Santiago it was an every day occurrence to have between one hundred and two hundred men report at sick call of a morning.

The chief diseases encountered were malaria, dysentery, typhoid, yellow fever or dengue, and thermic fever.

The everlasting sameness of the food was very trying. Coarse canned beef, canned tomatoes, fat bacon, and hardtack. The same thing three times a day, and day after day. The men got so that the very sight of the food would nauseate them, and if they took a few mouthfuls it would be immediately thrown up.

The men emaciated rapidly, and became so weak from fever and dysentery that they were not fit for the lightest duty. Just as sure as a detail were told off to get wood, dig a privy vault or to do any other work in the heat of the day, every one of them would be in the hospital with thermic fever.

Before leaving America, we had several cases of measles, and two developed on the transports on the way to Cuba. These were, however, immediately isolated and sent to the hospital ship, and we had no further trouble with it. The dysentery at one time began to assume alarming proportions. In the treatment of this we got better results from the subgallate of bismuth than with anything else we tried.

The most universal disease, however, was dengue fever. I do not suppose there were ten men in any one regiment who escaped it. Dr. Gonzales, the government yellow fever expert, who inspected all the hospitals, pronounced it yellow fever. If this was yellow fever, then that epidemic which prevailed all over the State of Texas about a year ago, and we called dengue, was all yellow fever. The symptoms were precisely similar. High temperature, sallow complexion, bilious vomiting, and pains all over the body, especially severe down the spine. There were six or eight regiments encamped around us, and they all had it. In these there were no deaths, although I believe there were a few deaths at Siboney, on the coast, where the base hospital was established. I never saw a case of marked jaundice, but in all there was bilious vomiting more or less severe. Neither did I see a single case of black vomit. Some had albumen in the urine, and others not.

Each one on entering the hospital would have a very high temperature, usually 105° or over. A large dose of quinine would invariably bring the temperature down to about normal by morning, and this effect was had so constantly as to argue a malarial origin. Suppression of urine was a frequent accompaniment; it and the intense pain in the small of the back were usually relieved by drinking large quantities of water.

The hospital accommodations were very bad indeed. Our regimental hospital consisted of a large sheet stretched across a pole. The patients all had to sleep on the ground, which was soaking wet. Every afternoon, just as regularly as the afternoon came around, there would be a downpour of rain. The ground, cut up by thousands of feet, was always ankle deep in mud.

The army was recalled from Cuba just in time; a week longer, and I do not think they would have been able to move us. The sickness at Montauk Point was just the development of fevers originating in Cuba.

In the First Volunteer Cavalry we had only two deaths from sickness; one from spinal meningitis while in San Antonio, and the other from dysentery on board the transport Miami while on the way home from Cuba.

